# Neurocognitive Impairment and Associated Factors Among Elderly in the Bahir Dar City Administration, Northwest Ethiopia

**DOI:** 10.3389/fnagi.2022.888704

**Published:** 2022-07-13

**Authors:** Betelhem Fekadu, Minale Tareke, Meseret Tadesse, Tamrat Anbesaw

**Affiliations:** ^1^Department of Psychiatry, College of Medicine and Health Science, Wollo University, Dessie, Ethiopia; ^2^Department of Psychiatry, College of Medicine and Health Science, Bahir Dar University, Bahir Dar, Ethiopia

**Keywords:** neurocognitive, prevalence, risk factors, elderly, Ethiopia

## Abstract

**Background:**

Neurocognitive impairment is a widely common problem in the elderly. It encompasses mild and major cognitive impairment, which will lead to disability and increase the risk of death. It also compromises the quality of life of the patient and poses a burden on the family and society as a whole. However, there is a paucity of information concerning neurocognitive impairment among the elderly in Ethiopia. This study aimed to assess the prevalence of neurocognitive impairment and associated factors among the elderly in Bahir Dar city, Ethiopia 2020.

**Methods:**

A community-based cross-sectional study was conducted among 423 respondents using a simple random sampling technique from 1 June to 30 June 2020. Neurocognitive impairment was measured using the Mini-Mental State Exam adjusted cutoff point (presence or absence) by the level of education of the participants. Data were entered into EpiData version 4.62 and exported to SPSS version 23 for analysis: descriptive statistics were used for summarization and presentation and binary logistic regression for a measure of association between exposures and outcome variable.

**Results:**

The prevalence of neurocognitive impairment was 42.1%. Factors such as having no spouse [AOR = 1.76, 95% confidence interval (CI): 1.08–2.86], having depression (AOR = 3.04, 95% CI: 1.80–5.14), lifetime alcohol use (AOR = 2.90, 95% CI: 1.19–7.07), and having low family support (AOR = 3.07, 95% CI: 1.35–6.96) and moderate family support (AOR = 1.83; 95% CI: 1.10–3.06) were significantly associated with neurocognitive impairment.

**Conclusion and Recommendation:**

The prevalence of neurocognitive impairment was high in Bahir Dar city administration. Neurocognitive impairment has shown significant association with no spouse, depression, alcohol use, and low and moderate family social support. It is important to pay attention to old age with comorbid mental illness and also to strengthen social support systems to prevent and manage neurocognitive impairment.

## Introduction

Neurocognitive impairment is a clinical syndrome caused by neurodegeneration and characterized by a progressive deterioration in cognition and capacity for independent living. It affects many cognitive domains, including memory, thinking, language function, visual function, comprehension, and judgment (Diagnostic, 2013). The deterioration can be caused by a number of brain disorders or injuries that primarily or secondarily affect the brain, such as Alzheimer’s disease or stroke, and the cognitive impairment is commonly accompanied by a decline in emotional control, social behavior, or motivation (World Health Organization [WHO], 2012).

Life expectancy is increasing across the world, with population aging rapidly increasing in low-income and middle-income countries (LMICs), where the prevalence of cognitive impairment is therefore expected to increase (Prince et al., 2013b). Cognitive impairment is one of the non-communicable diseases expected to be a very devastating problem as it is highly deteriorating the quality of life of the elderly, which makes them unable to perform daily basic activities, increases the risk of mortality and morbidity, and is the major cause of dependency among the elderly worldwide (Sousa et al., 2009; Scotland, 2011).

The impact of neurocognitive impairment goes far beyond those with the condition, as it also affects their families on personal, emotional, social, and financial levels, resulting in poverty in many households, and making dementia a global health priority (Thies and Bleiler, 2013; Kochanek et al., 2019). Worldwide, neurocognitive is estimated to affect 47 million people; it is believed that the number will increase to 75 million by the year 2030, and will be threefold by 2050. Among those, 57% of them lived in LMICs, and this number is expected to rise to 71% by the year 2050 (Prince et al., 2013a). In Ethiopia, there were more than 4.2 million older adults in 2013 who were more than 60 years of age, and it is assumed to become 5.2 million by the year 2025 (Central statistical Agency [Ethiopia], 2014).

Neurocognitive impairment is largely underdiagnosed, and often at the time of diagnosis, patients are at a late stage in the disease process (Prince et al., 2015). According to the World Alzheimer Report, many people living with cognitive impairment have not received a formal diagnosis and treatment. This is more prominent in LMICs, where most of the population has a low level of education (Batsch and Mittelman, 2012); due to this, many of those affected individuals are delayed in seeking medical care and have poor outcomes (Yusuf and Baiyewu, 2012). According to numerous studies discussed, the prevalence of cognitive impairment in older adults (aged 60 years and above) in India was 26.06%, China 13.6%, and Malaysia 36.5% (Al-Jawad et al., 2007; Han et al., 2019; Mohan et al., 2019). While in Jamaica, 21.2% of older adults had mild cognitive impairment and 11.0% had severe impairment (Waldron et al., 2015), Iran 7.9% (Sharifi et al., 2016), Taiwan 65.6 (Chen et al., 2017), and Saudi Arabia 45% (Alkhunizan et al., 2018). Furthermore, a community-based study in Benin, Cameroonians, and South Africa showed that the prevalence of cognitive impairment was 3.7, 33.3, and 16.9%, respectively (Paraïso et al., 2011; Ramlall et al., 2013; Tianyi et al., 2019).

Also, many studies have shown a link between cognitive impairment and various risk factors such as female gender, single status, advanced age, lower levels of education, lower socioeconomic status, living alone, increased physical health problems [such as diabetes mellitus (DM), stroke, hypertension (HTN), and hearing loss], head injury, presence of depression, anxiety, use of lipid-lowering drugs, dependence in one or more activities of daily living, physical inactivity, smoking cigarette, and alcohol consumption. Those who were associated positively with neurocognitive disorder (NCD) were more likely to have cognitive impairment (Ramlall et al., 2013; Waldron et al., 2015; Konda et al., 2018; Ren et al., 2018a,b; Bich et al., 2019; Han et al., 2019; Mohan et al., 2019; Tianyi et al., 2019).

Even though there is a high prevalence and burden of the condition in this particular age group in LMICs, it is not well assessed and planned for the possible intervention program in health policy and strategies of Ethiopia. To the best of our knowledge, there are no studies that have reported the prevalence and associated factors of neurocognitive impairment among the elderly in Ethiopia. Thus, this study aimed to determine the prevalence and identify factors associated with neurocognitive impairment in older adults. This study will help policymakers, future researchers, and health planners, and serve as a foundation for future public health and mental health research in the area. Additionally, it contributes to the development of knowledge about neurocognitive impairments among the elderly population.

## Materials and Methods

### Study Area

The study was conducted in the Bahir Dar city administration, which is located 565 km north of Addis Ababa in Ethiopia’s northwestern area and is the capital of the Amhara regional state. According to the 2016–2017 city administration report, Bahir Dar has a total population of 266,952 people, with 124,396 men and 142,555 women. There are 9 sub-cities in the city, with a total of 66,628 households. Among them, the number of people aged 60 years and above is predicted to be 11,034 (5,003 men and 6,031 women). From the sub-city, there are 1,670 older adults in Shimbit, 1,043 in Tana, 1,200 in Fasilo, 287 in Sefene Selam, 522 in Gish Abay, 417 in Shum Ambo, 1,591 in Belay Zeleke, and 4,304 in Ginbot-20. Two specialized hospitals and four primary private hospitals provide health treatment to these people. The information was obtained from the Bahir Dar city municipality.

### Study Design and Period

A community-based, cross-sectional study was conducted from 1 June to 30 June 2020.

### Source Population

The source of this study comprises the elderly in the Bahir Dar city administration.

### Study Population

All old age populations in Bahir Dar city administration during the study period.

#### Sampling Unit

All households were included in the sample.

#### Study Unit

Individual elderly from whom data were collected in Bahir Dar city administration.

### Eligibility Criteria

The participants who provided consent were those aged 60 and above, literate (grade 5 and above), and residents of the town for at least 6 months were included. Those who have visual or hearing problems (as they would be unable to answer visuospatial questions) and are severely ill and unable to respond were excluded.

### Sample Size Determination

The sample size is calculated by using the Epi info 7 StatCalc software by considering the following assumptions: expected frequency of 50%, as there was no previous study done in Ethiopia; the confidence limit = 5%. Therefore, *n* = 384, as the population size is greater than 10,000, there is no need for a correction factor. By adding a 10% non-response rate, the final sample size was 423.

### Sampling Method and Technique

There are nine sub-cities in the Bahir Dar city administration urban division, one of which (Hidar 11) was left out of the report due to a lack of data. The final sample size was proportionally distributed to eight sub-cities, namely, Shimbit (64), Tana (40), Fasilo (46), Sefene Selam (11), Gish Abay (20), Shum Abo (16), Belay Zeleke (61), and Gimbot Haya (165), based on the population size. Health extension workers provided the sample frame (households with varying ages), and each household was then randomly picked using the lottery method. If a house has more than one member who meets the criterion, a lottery was used. If none of the participants in the chosen family met the criteria, the next household was chosen.

### Operational Definition

#### Neurocognitive Impairment

Mini-Mental State Examination (MMSE) score of ≤22 for those who attended less than eighth grade, ≤24 for those who attended grade 9–12, and ≤26 for those college/university graduates out of a total score of 30 (Gugssa et al., 2011).

#### Depression

Score of 5 and above using the Geriatric Depression Scale (GDS) (Sadock and Sadock, 2011).

#### Quality of Life

Using the WHOQOL-BREF 26-item index, a higher score denotes a better quality of life (da Rocha et al., 2012).

#### Activities

A total score of 6 indicates independence, 4 indicates moderate independence, and 2 or less indicates dependence, according to Katz’s daily life independence indicator.

(Wallace and Shelkey, 2007).

#### The Multidimensional Scale of Perceived Social Support Scale

Any mean scale score ranging from 1 to 2.9 was considered as low social support, 3 to 5 as moderate social support, and 5.1 to 7 as high social support (Zimet et al., 1988).

#### Current Substance Use

Using one of the specific substances for non-medical purposes within the last 3 months (alcohol, khat, tobacco, and others) (World Health Organization [WHO], 2016).

#### Ever Use of a Substance

At least once in a lifetime, using at least one of any specific substances for a non-medical purpose (alcohol, khat, tobacco, etc.) (World Health Organization [WHO], 2016).

#### The Elderly

Aged 60 and above (World Health Organization [WHO], 2020).

#### Fast Alcohol Screening Test

A total score of 3 shows that you are using alcohol in a hazardous manner (Hodgson et al., 2002).

#### Nutritional Status

Using the Mini Nutritional Assessment, which has a score range of 0–14 and can be interpreted as malnourished (0–7) or at risk of malnutrition (8–11) or normal nutritional status (12–14) (Hailemariam et al., 2016).

#### Income

Low income was defined as individuals with an average monthly income of less than 2,004 ETB (US$1.9/day) using the World Bank poverty line cut point of US$1 = 35.16 ETB (Lee, 2019).

### Data Collection Tools

A structured interviewer-administered questionnaire was used, which has seven subsections, namely, sociodemographic questionnaire to assess the patients’ background information, including age, sex, marital status, language, educational status, income, religion, and employment status. History of mental illness and presence of chronic medical illness will be assessed with yes/no questions. Yes/no questions were used to assess substance-related factors, but alcohol drinking was assessed by using Fast Alcohol Screening Test (FAST). Neurocognitive impairment was measured by using a standardized MMSE. It has 5 domains of functions and each has its own score: the orientation domain has 10 scores, registration out of 3, attention/calculation has 2 components: serial of 7 for literate and word reversal for less educated. Other domains such as recall, language, and praxis were included, which have a total score out of 30. The cutoff point was modified for participants based on their level of education. It has been validated in Ethiopia for a population who have formal education grades fifth and above with respective cutoff points modified based on the level of education (Gugssa et al., 2011). The specificity and sensitivity of MMSE were 77.8 and 78.7%, respectively (Folstein et al., 1975). Depression was assessed using the 15 items of the short form GDS, which were found to have 92% sensitivity and 89% specificity when evaluated against the diagnostic criteria. The validity and reliability of the tool have been supported through both clinical practice and research (Sheikh and Yesavage, 1986). Quality of life was assessed using WHOQOL-BREF. It has four domains, which are physical, psychological, social, and environmental. It was translated into nine languages and has an internal consistency (Cronbach’s alpha 0.70) (da Rocha et al., 2012). Mini-nutritional assessment short form has six questions and has been validated in Ethiopia. Cronbach’s alpha was 0.65; sensitivity was 80.1% and specificity was 72.5% (54). Katz’s index of independence in activities of daily living has six questions each with one point. The validity of Katz was found to have Cronbach’s alpha of 0.83 and test-retest and inter-rater reliability was excellent (55). The English version of the instruments was translated to the local language by an independent language expert and retranslated again into English by experienced mental health professionals.

### Data Collection Procedures

Data were collected through an interview by trained data collectors. Four psychiatric nurses were recruited for data collection, and one mental health specialist was recruited for supervision. The training was given to data collectors and supervisors by the principal investigator on the methods of data collection and the detail of the questionnaire. The principal investigator checked for the completeness of the data.

### Data Quality Control

To assure the data quality, high emphasis was given to designing data collection instruments. Structured questionnaires were used for final data collection. A standardized questionnaire was translated into local languages Amharic by the language experts, and then it was translated back to English by an independent person to check for consistency and understandability of the tool, and a pretest was conducted on 5% (21 individuals) from Hidar 11 community out of the study area for clarity of questionnaires and the items of questions were modified accordingly. Prior to data collection, training and supervision were undertaken throughout the data collection period to make the data consistent. The questionnaire was checked for its completeness on a daily basis by data collectors, supervisors, and the investigator, for the incomplete data, which were traced back and completed again.

### Data Management, Analysis, Interpretation, and Presentation

Data were entered into EpiData version 4.6 and transferred to SPSS version 23 for analysis. Descriptive statistics were used to describe the data. Binary logistic regression analysis was utilized to assess the association between independent and dependent variables by calculating the AOR and their 95% CIs. All variables with a *p*-value of less than or equal to 0.2 in the bivariate analysis were included in the multivariate analysis. Then, variables with a *p*-value of <0.05% were considered statistically significant.

## Results

### Sociodemographic Characteristics of Respondents

A total of 423 study participants were included, giving a response rate of 100%. The median age of the respondents was 65 years with an interquartile range of 8. Among participants, 249 (58.9%) were men. Two-thirds of the participants, i.e., 282 (66.7%) were Orthodox Christian, half of the respondents, i.e., 221, were married (52.2%), and 365 (86.3%) were Amhara in ethnicity. Regarding educational level, 260 (61.5%) were from grades 5 to 8. Around one-third of them, i.e., 155 (36.6%) were housewives, followed by 114 (27%) retired people (Table 1).

**TABLE 1:** Sociodemographic characteristics of respondents in the Bahir Dar City administration, Bahir Dar, Ethiopia, 2020 (*n* = 423).

Variable	Catagories	Frequency	Percentage (%)
Sex	Female	174	41.1
	Male	249	58.9
Age	60–64	191	45.1
	65–69	127	30
	70–74	69	16.3
	75–79	18	4.3
	80 and above	18	4.30
Religion	Orthodox	282	66.6
	Muslim	117	27.7
	Protestant	16	3.80
	Catholic	8	1.90
Marital status	Has spouse	221	52.2
	No spouse*	202	47.8
Occupation	Employed	17	4
	Merchant	105	24.8
	Farmer	2	0.5
	Day laborer	26	6.2
	Housewife	155	36.6
	Jobless	4	0.9
	Retired	114	27
Educational status	5–8th grade	260	61.5
	9–12th grade	99	23.4
	College and above	64	15.1
Ethnicity	Amhara	365	86.3
	Oromo	14	3.3
	Tigray	27	6.4
	Gurage	17	4
Average monthly income	<2004 ETB	157	37.1
	≥2004 ETB	266	62.9
Current living condition	Alone	92	21.7
	Relative	269	63.6
	Family	62	14.7

*Divorced, separated, and widowed.

### Physical Health of the Respondents

Regarding the physical health of the respondents, 51.31% of them had a medical illness. Of these, 59.9% had HTN, followed by DM (35%). Among the participants, 46.8% used medications for their illnesses (Table 2).

**TABLE 2:** Description of physical health of the respondents in the Bahir Dar City administration, Bahir Dar, Ethiopia, 2020 (*n* = 423).

Variable	Catagories	Frequency	Percentage (%)
Presence of medical illness	Yes	217	51.31
	No	206	48.69
Hypertension	Yes	130	59.9
	No	87	40.1
Diabetes mellitus	Yes	76	35
	No	141	65
Cardiac problem	Yes	25	11.5
	No	192	88.5
HIV/AIDS	Yes	26	11.9
	No	191	88.1
Current medication	Yes	198	46.8
	No	225	53.2
Hx of head trauma	Yes	50	11.8
	No	373	88.2
Nutritional status	Malnourished	32	75.6
	Risk	165	39
	Normal	226	53.4

### Substance-Related Factors of the Respondents

**FIGURE 1:**
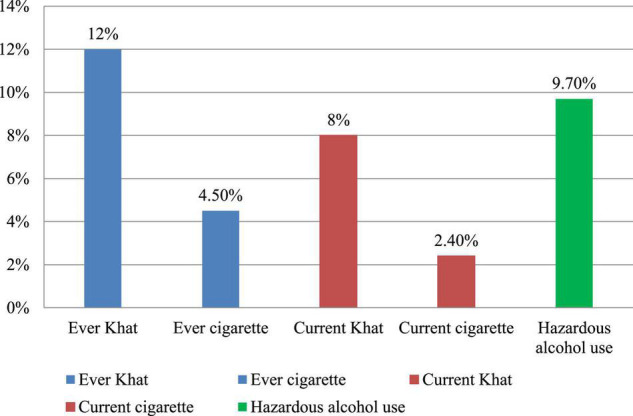
Ever and current substance use of participants in the Bahir Dar City administration, Bahir Dar, Ethiopia, 2020.

Of the total respondents, 9.7% of them were hazardous alcohol users, 8% of them used khat, and 2.4% used cigarettes in the past 3 months (Figure 1).

### Mental and Psychosocial Characteristics of Respondents

Notably, 57.9% of respondents had depression, and 47.5% had a poor quality of life (Table 3).

**TABLE 3:** Mental and psychosocial characteristics of participants in the Bahir Dar City administration, Bahir Dar, Ethiopia, 2020 (*n* = 423).

Variable	Catagories	Frequency	Percentage (%)
Family history of mental illness	Yes	34	8
	No	389	92
Depression	Yes	245	57.9
	No	178	42.1
Significant other social support	Low	57	13.5
	Moderate	150	35.5
	High	216	51
Family social support	Low	55	13
	Moderate	136	32.2
	High	232	54.8
Friend social support	Low	56	13.2
	Moderate	213	50.4
	High	154	36.4
Quality of life	Poor	201	47.5
	High	222	52.5
The activity of daily living	Dependence	9	2.1
	Moderate dependence	14	3.3
	Independent	400	94.6

### Prevalence of Neurocognitive Impairment

In this study, 178 (42.1%) respondents had neurocognitive impairment [95% confidence interval (CI): 37.5–46.7]. Of these, 105 (59%) were women and 73 (41%) were men.

### Factors Associated With Neurocognitive Impairment Among Elderly

Bivariable logistic regression variables, including above 80 years of age, presence of the medical condition, having no spouse, lower income, living with a relative, family history of mental illness, current medication use, malnutrition, depression, hazardous alcohol use, poor quality of life, low social support from family, significant others, and friends, were found to have a *p*-value of less than 0.2. These variables fulfilled the minimum requirements for further multivariable logistic regression.

Multivariable logistic regression variables, including depression, hazardous alcohol use, having no spouse, and low and moderate family social support, were found to have a statistically significant association with neurocognitive impairment at a *p*-value of less than 0.05. Participants who had no spouse were 1.76 times (AOR = 1.76, 95% CI: 1.08–2.86) more likely to develop neurocognitive impairment than those who had a spouse. The odds of having neurocognitive impairment among respondents who had depression were three times as compared to those without depression (AOR = 3.04, 95% CI: 1.80–5.14).

In this study, the odds of having neurocognitive impairment among respondents who used alcohol were three times higher as compared to those who did not use alcohol (AOR = 2.9, 95% CI: 1.19–7.07). The odds of having neurocognitive impairments who had low and moderate social support from the family were three times (AOR = 3.07, 95% CI: 1.35–6.96) and 1.83 (AOR = 1.83; 95% CI: 1.10–3.06) as compared to the one with high family social support (Table 4).

**TABLE 4:** Bivariable and multivariable binary logistic regression analysis showing the association between neurocognitive impairment and associated factors among elderly in Bahir Dar City administration, Bahir Dar, Ethiopia, 2020 (*n* = 423).

Explanatory variables	Neurocognitive impairment		COR (95% CI)	AOR (95% CI)	P-value
	Yes	No			
Age					
60–64	75	116	1	1	
65–69	47	80	0.909 (0.572, 1.44)	0.76 (0.43, 1.32)	0.336
70–74	36	33	1.68 (0.969, 2.65)	1.41 (0.72, 2.75)	0.313
75–79	7	11	0.984 (0.36, 2.65)	0.90 (0.26, 3.10)	0.869
80 and above	13	5	4.02 (1.37, 11.74)	1.00 (0.29, 3.46)	0.996
Marital status					
Has spouse	117	85	1	1	0.022
No spouse	61	160	3.61 (2.40, 5.42)	1.76 (1.08, 2.86)*	
Living condition					
Alone	55	37	3.218 (1.97, 5.25)	1.25 (0.62, 2.53)	0.527
Relative	38	24	3.427 (1.93, 6.07)	1.69 (0.79, 3.61)	0.171
Family	85	184	1	1	
Income					
<2004	79	78	1.7 (1.14, 2.54)	1.11 (0.67, 1.83)	0.681
≥2004	99	167	1	1	
Medical condition					
Yes	103	113	1.6 (1.08, 2.36)	1.5 (0.51, 4.45)	0.458
No	75	132	1	1	
Current medication use					
Yes	100	98	1.923 (1.3, 2.84)	1.55 (0.97, 2.47)	0.063
No	78	147	1	1	
Family history of mental illness					
Yes	23	11	3.157 (1.49, 6.66)	1.64 (0.70, 3.84)	0.248
No	155	234	1	1	
Nutritional status					
Malnourished	23	9	6.612 (2.90, 15.06)	2.35 (0.94, 5.84)	0.066
Risk	92	73		1.63 (0.99, 2.68)	0.052
Normal	63	163	3.261 (2.136, 4.97) 1	1	
Alcohol use					
Yes	30	11	4.31 (2.09, 8.86)	2.90 (1.19, 7.07)*	0.019
No	148	234	1	1	
Depression					
Yes	140	105	4.91 (3.16, 7.61)	3.04 (1.80, 5.14)*	
No	38	140	1	1	<0.0001
Quality of life					
Poor QOL	120	81	4.18 (2.77, 6.32)	1.59 (0.96, 2.61)	0.067
Good QOL	58	164	1	1	
Significant others’ support					
Low	42	15	6.95 (3.59, 13.44)	0.73 (0.16, 3.19)	0.681
Moderate	74	76	2.41 (1.56, 3.73)	1.04 (0.49, 2.21)	0.902
High	62	154	1	1	
Family support					
Low	42	13	7.95 (4.01, 15.76)	3.07 (1.35, 6.96)*	0.007
Moderate	69	67	2.53 (1.63, 3.93)	1.83 (1.10, 3.06)* 1	0.019
High	67	165	1		
Friend support					
Low	42	14	7.74 (3.84, 15.59)	2.00 (0.69, 5.77)	0.198
Moderate	93	120	2.0 (1.28, 3.11)	1.39 (0.80, 2.41)	0.238
High	43	111	1		

## Discussion

To the best of our knowledge, this is the first study to determine the prevalence and associated factors of neurocognitive impairment among the elderly population in Ethiopia. The result of this study indicated that the prevalence of neurocognitive impairment among the elderly was 42.1% (95% CI, 37.5–46.7). This was comparable with another study conducted in Saudi Arabia, which indicated that the prevalence was 45% (Alkhunizan et al., 2018). In contrast, the prevalence of neurocognitive impairment in this study is lower than in the study conducted in Taiwan, i.e., 65.6% (Chen et al., 2017). The possible reason for this discrepancy might be the difference in sample size and age of participants. In Taiwan, 946 participants had a neurocognitive impairment and were aged 80 years and above. The other possible reason might be the inclusion of individuals with an educational level of less than grade six in the prior study without adjusting the cutoff point. Furthermore, the difference in participants who had different socioeconomic and demographic characteristics in the populations might be another possible explanation for their variation.

However, in some other studies, the proportion of neurocognitive impairment was lower than in this study, i.e., 26.06% in a study conducted in India (Mohan et al., 2019), 13.6% in China (Han et al., 2019), 7.9% in Iran (Sharifi et al., 2016), 33.3% in Cameroon, and 16.9% in South Africa (Ramlall et al., 2013; Tianyi et al., 2019). This variation might be due to the difference in screening tool used; in a previous study, Addenbrooke’s cognitive examination tool was used in India (Mohan et al., 2019) and the Brief Cognitive Assessment Tool (BCAT) was used in Iran (Sharifi et al., 2016). In contrast, in this study, MMSE was utilized. The inclusion criteria might be another possible reason for their incongruence; in the Cameroonian study, participants in the age group of 50 years and above were included; as it is clear that impairment is associated with age, in most of the studies, the lesser is the age, the lesser cognitive impairment may occur (Tianyi et al., 2019). Also, the difference in sociodemographic characteristics such as culture and health-seeking behavior of the participants might be another reason for the variation.

Regarding the associated factors, this finding also revealed that those who had no spouse (separated, widowed, and divorced) were 1.76 times more likely to develop neurocognitive impairment as compared to those who had a spouse. This finding was in agreement with studies from China (Ren et al., 2018b) and Cameroon (Tianyi et al., 2019). The possible reason might be due to less social engagement because of the loss of their spouse and associated depression, as relationship helps in the social development of an individual (Yaffe, 2009).

This study showed that elders who had depression were three times more likely to develop cognitive impairment than undepressed participants. These findings were similar to different studies from India (Mohan et al., 2019), Jamaica (Waldron et al., 2015), and Brazil (Winter Holz et al., 2013). Also, there is a strong association between depressive symptoms and cognitive impairment. The possible explanation might be that depression serves as an early sign or a risk factor for the development of neurocognitive impairment, especially if not treated. Another reason could be the presence of pseudodementia, a condition where individuals who had a mental illness, especially depression, might overestimate the association between neurocognitive impairment and depression.

Elders who had low family social support were nearly three times and those who had moderate family social support were nearly two times more likely to develop neurocognitive impairment as compared to those who had strong family social support. This finding was in agreement with a study from Malaysia (Al-Jawad et al., 2007). The possible explanation for the association could be people who receive good baseline social support, especially from the family, had a better cognitive outcome. Social and good family integration lessen psychological stress and even improve physical health, which can prevent cognitive decline. The more people involve in social activities and receive family support, the more they become exposed to different stimuli to improve their cognitive ability; on the contrary, the more people lack social support, especially from family members, the more the risk of having cognitive impairment (Han et al., 2019).

Finally, in this study, the elderly who use alcohol throughout their lifetime were three times more likely to develop neurocognitive impairment, as supported by the studies in Taiwan (Chen et al., 2017) and India (Mohan et al., 2019). This might happen because alcohol will prevent the regeneration of neurons and cause deterioration of cognitive ability as well as affect memory formation, visuospatial capacity, and executive function due to multiple brain lesions. There will be premature aging of the brain in the elderly because alcohol could induce brain atrophy. In addition, as alcohol may lead to other physical and mental problems, it can indirectly create cognitive impairment (Bernardin et al., 2014).

### Limitations of the Study

The generalizability of the study might be limited for those who had formal education, as the tool (MMSE) is adapted based on the educational level in this setup. Age was taken only subjectively where there was no recorded birth certificate. Other limitations include that some of the reports were based on prior events, which can lead to recall bias.

## Conclusion

In this study, the prevalence of neurocognitive impairment was high as compared to studies conducted in sub-Saharan countries. Those who have neurocognitive impairment were shown to have a significant association with no spouse, depression, low and moderate family social support, and alcohol use. It will be better if health extension workers screen and link the elderly with cognitive decline to hospitals for early management. Also, information about the need for social support for the elderly should be provided to caregivers and both families, and the elderly should be educated on the consequence of alcohol on health and early sign of cognitive impairment.

## Data Availability Statement

The original contributions presented in this study are included in the article/supplementary material, further inquiries can be directed to the corresponding author.

## Ethics Statement

The studies involving human participants were reviewed and approved by Bahir Dar University Ethical Institutional Review Board. The patients/participants provided their written informed consent to participate in this study.

## Author Contributions

BF, MeT, MiT, and TA contributed to the conception, design, and revising of the work for important intellectual content. BF and TA contributed to the analysis in drafting and interpretation of data. TA participated in framing the method and wrote the manuscript. All authors have read and approved the final manuscript.

## Conflict of Interest

The authors declare that the research was conducted in the absence of any commercial or financial relationships that could be construed as a potential conflict of interest.

## Publisher’s Note

All claims expressed in this article are solely those of the authors and do not necessarily represent those of their affiliated organizations, or those of the publisher, the editors and the reviewers. Any product that may be evaluated in this article, or claim that may be made by its manufacturer, is not guaranteed or endorsed by the publisher.

## Abbreviations

CI: confidence interval
DM: diabetes mellitus
EPI INFO: epidemiological information
FAST: Fast Alcohol Screening Test
GDS: Geriatric Depression Scale
HTN: hypertension
LMICs: low- and middle-income countries
MMSE: Mini-Mental State Examination
NCD: neurocognitive disorder
OSSS: Oslo Social Support Scale
SPSS: Statistical Package for Social Science
SSA: sub-Saharan African
US: United Sate
WHO: World Health Organization
WHOQOL-BREF: World Health Organization Quality of Life Scale
YLDs: years lived with disability.
